# Modeling network phenomena in the Inferior Olive: I. Keeping track of time

**DOI:** 10.1186/1471-2202-12-S1-P379

**Published:** 2011-07-18

**Authors:** Merav Stern, Benjamin Torben-Nielsen, Yaara Lefler, Idan Segev, Yosef Yarom

**Affiliations:** 1 Edmond and Lily Safra Center for Brain Sciences, Hebrew University, Jerusalem, Israel; 2 Interdisciplinary Center for Neural computation, Hebrew University, Jerusalem, Israel; 3 Department of Neurobiology, Hebrew University, Jerusalem, Israel

## 

Output spikes of the Inferior Olive (IO) are of great importance to cerebellar function because they directly trigger complex spikes in the Purkinje cells and/or provides timing framework for cerebellar function. There is ample evidence that the exact timing of IO output relates to underlying sub-threshold oscillations (STOs). Thus, the IO can be seen as an internal clock and "timing" can be related to the frequency and the exact phase of the STOs.

In this work we investigated how IO (sub)networks can keep track of time under the presence of perturbations such as spikes or currents arriving from gap-junction coupled cells. We define “keeping track of time” as the ability of the IO cells to maintain a zero-phase shift after a perturbation. Individual cells are assumed to be intrinsic oscillators which spontanuously oscillate due to the interaction between a low-threshold calcium (T-type) current and a leak current [1].

First, we numerically simulated single neurons and small networks consisting of (at least) twocells coupled by gap-junctions (using NEURON) and injected various brief current pulses at all phases of the STO to elecit a voltage deflection in one cell. the resultant phase shift of the next peak was measured. This way we found that all voltage deflections larger than 1 mV (except when occurring at the peak of the STO) caused the phase to shift in single cells. Moreover, a spike in one cell would cause a phase shift in all gap-junction coupled cells as well. Second, we validated these results analytically using the phase-model formalism, as in [2]. We analytically established that small perturbations always shift the phase of the oscillation in a single cell. Moreover, we proved that a phase shift in one cell will also cause a phase shift in any gap-junction coupled cell; hence the phase of all STOs in connected cells will be shifted.

Thus, we showed that networks of gap-junction coupled intrinsic oscillators cannot act as a clock. Because this conclusion is contrary to evidence from *in-vitro* experiments in which no phase shift is observed after perturbations, we hypothesized that the STOs in the IO network are not caused “intrinsically” but emerge as a network phenomena. This type of “network oscillation” arises from a periodic current flowing trough the gap-junction as a consequence of heterogeneity in equilibrium potentials of the individual cells due to difference in the leak and low-threshold calcium densities[1]. We tested this hypothesis and found that a zero-phase shift is maintained throughout the network after a perturbation in one cells. Therefore, we conclude that the STO in the IO is actually a network effect and not an intrinsic property of all IO cells.

## References

[B1] Manor Y Rinzel J Segev I Yarom Y Low Amplitude Oscillations in the Inferior Olive: A model based on electrical coupling of neurons with heterogeneous channel densities J. Neurophysiol 1997 77 2736 2752 9163389 10.1152/jn.1997.77.5.2736

[B2] Ko TW Ermentrout GB Phase-response curves of coupled oscillators Phys. Rev. E 2009 79 016211 10.1103/PhysRevE.79.016211 19257126

